# Gone With the Windsock Diverticulum: An Incidentally Discovered Intraluminal Duodenal Diverticulum

**DOI:** 10.7759/cureus.103562

**Published:** 2026-02-13

**Authors:** Pierce L Claassen, Francisco C Ramirez, Terry L Jue

**Affiliations:** 1 Department of Internal Medicine, Mayo Clinic, Scottsdale, USA; 2 Division of Gastroenterology and Hepatology, Mayo Clinic, Scottsdale, USA

**Keywords:** developmental anomaly, duodenum defect, intraluminal duodenal diverticulum, rare cause of diarrhea, upper endoscopy

## Abstract

Intraluminal duodenal diverticulum (IDD) is an exceedingly rare congenital anomaly of the foregut. This entity is most frequently discovered incidentally during esophagogastroduodenoscopy; however, patients have reported symptoms of gastrointestinal obstruction, bleeding, or pancreatitis. In this report, we discuss a case of an IDD that was diagnosed during routine upper endoscopy in an adult woman who was referred to the gastroenterology clinic because of unexplained changes in her bowel habits.

## Introduction

Intraluminal duodenal diverticulum (IDD) may be incidentally discovered during routine esophagogastroduodenoscopy (EGD). IDD has an estimated prevalence of less than 0.05% [1]. IDD is a postulated congenital anomaly that arises in the seventh week of embryonic development due to aberrant recanalization of a duodenal diaphragm. Patients most frequently present with epigastric pain secondary to pancreatitis, bleeding, or duodenal obstruction [2]. Our case highlights an IDD that was diagnosed during EGD in a patient with changes in bowel habits.

This case was previously presented at the American College of Gastroenterology 2025 Annual Scientific Meeting, Phoenix, Arizona.

## Case presentation

**Figure 1:**
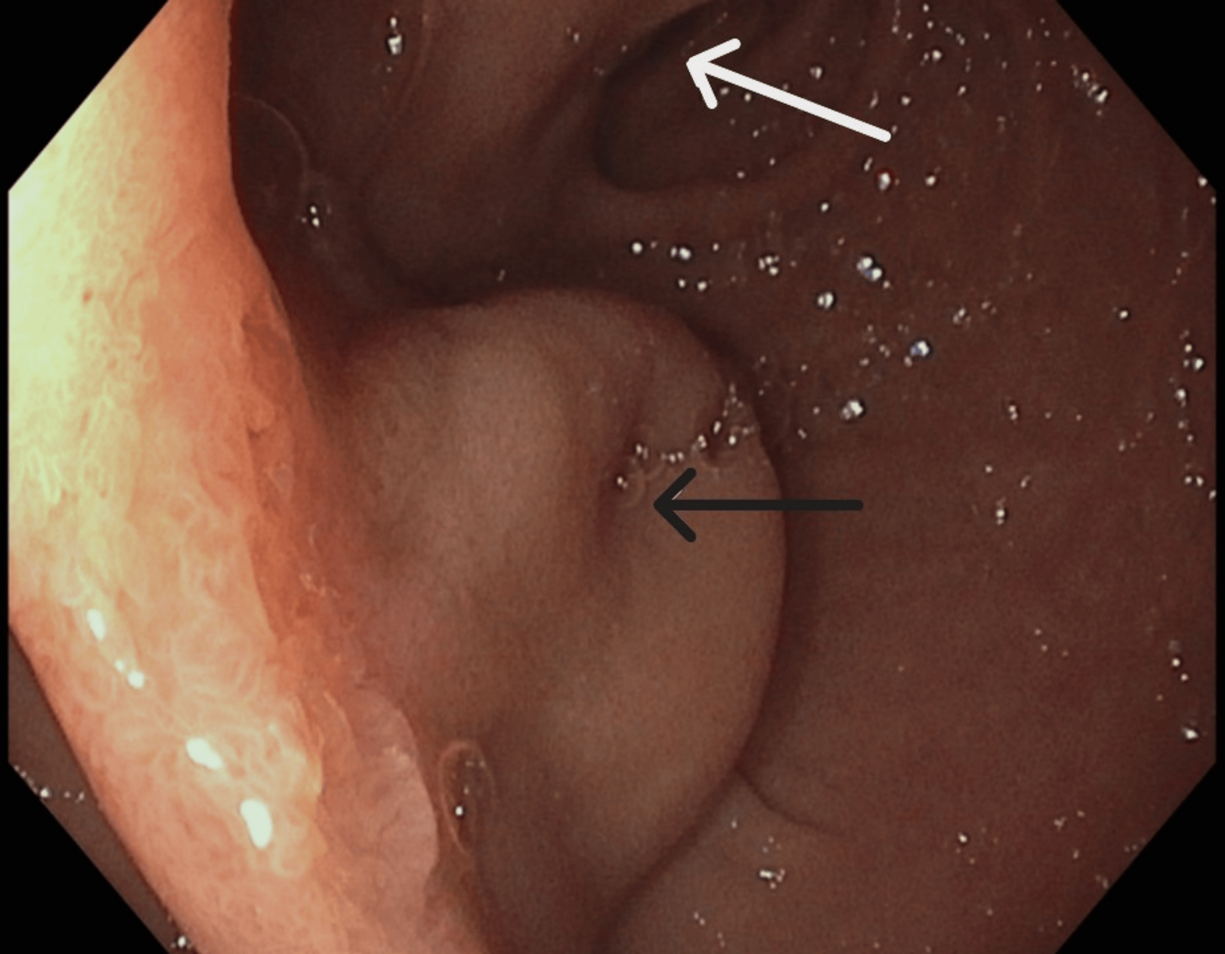
Endoscopic image of the intraluminal duodenal diverticulum in the foreground. The dome is positioned in the center with a partially indurated aperture (black arrow). The distal aspect of the intraluminal duodenal diverticulum extends distally into the true lumen of the duodenum (white arrow), as seen in the background of the image.

**Figure 2:**
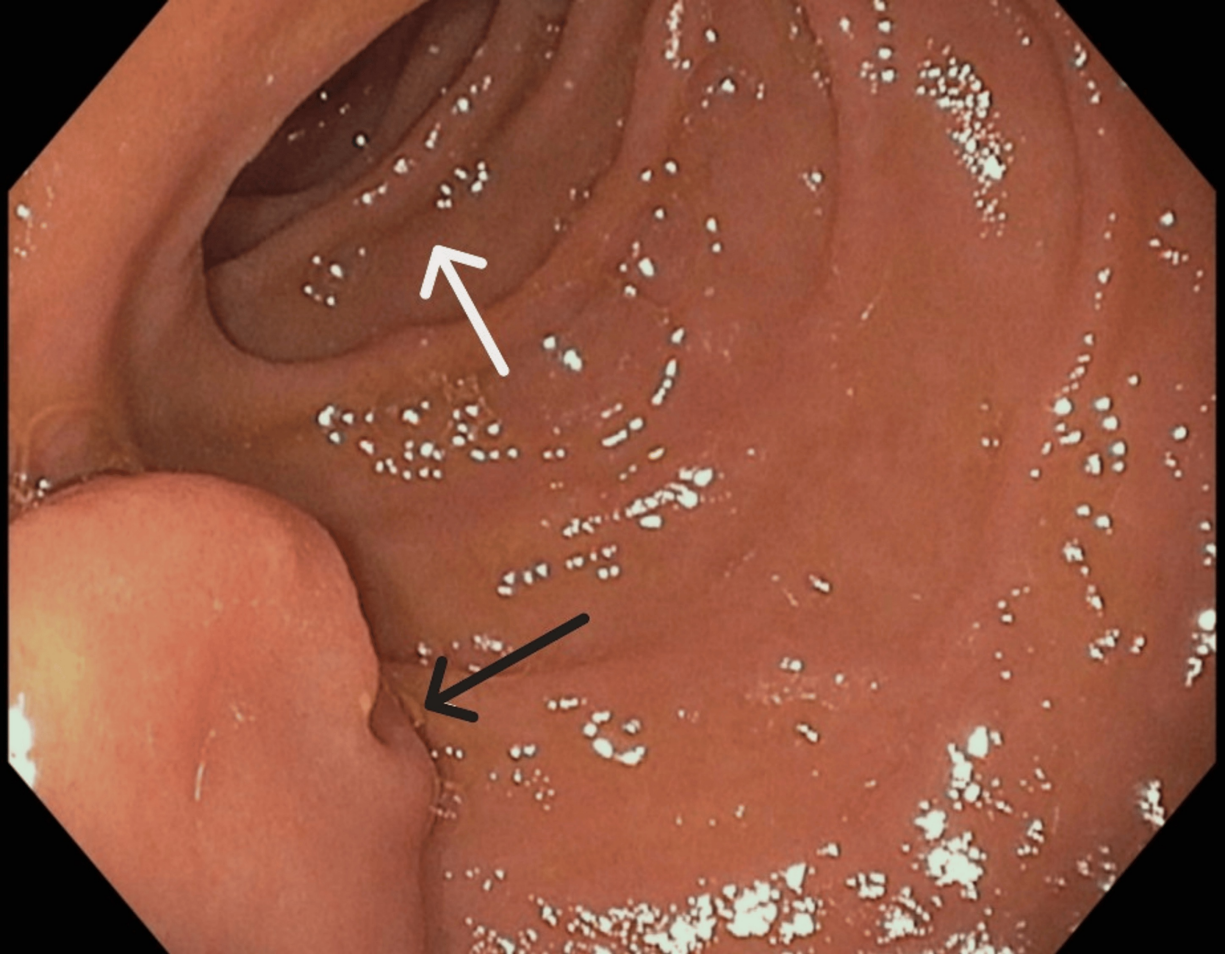
Endoscopic image of the notched aperture of the intraluminal duodenal diverticulum (black arrow) in the foreground of the left lower quadrant of the image. The second portion of the true lumen of the duodenum (white arrow) is seen in the background of the image.

**Figure 3:**
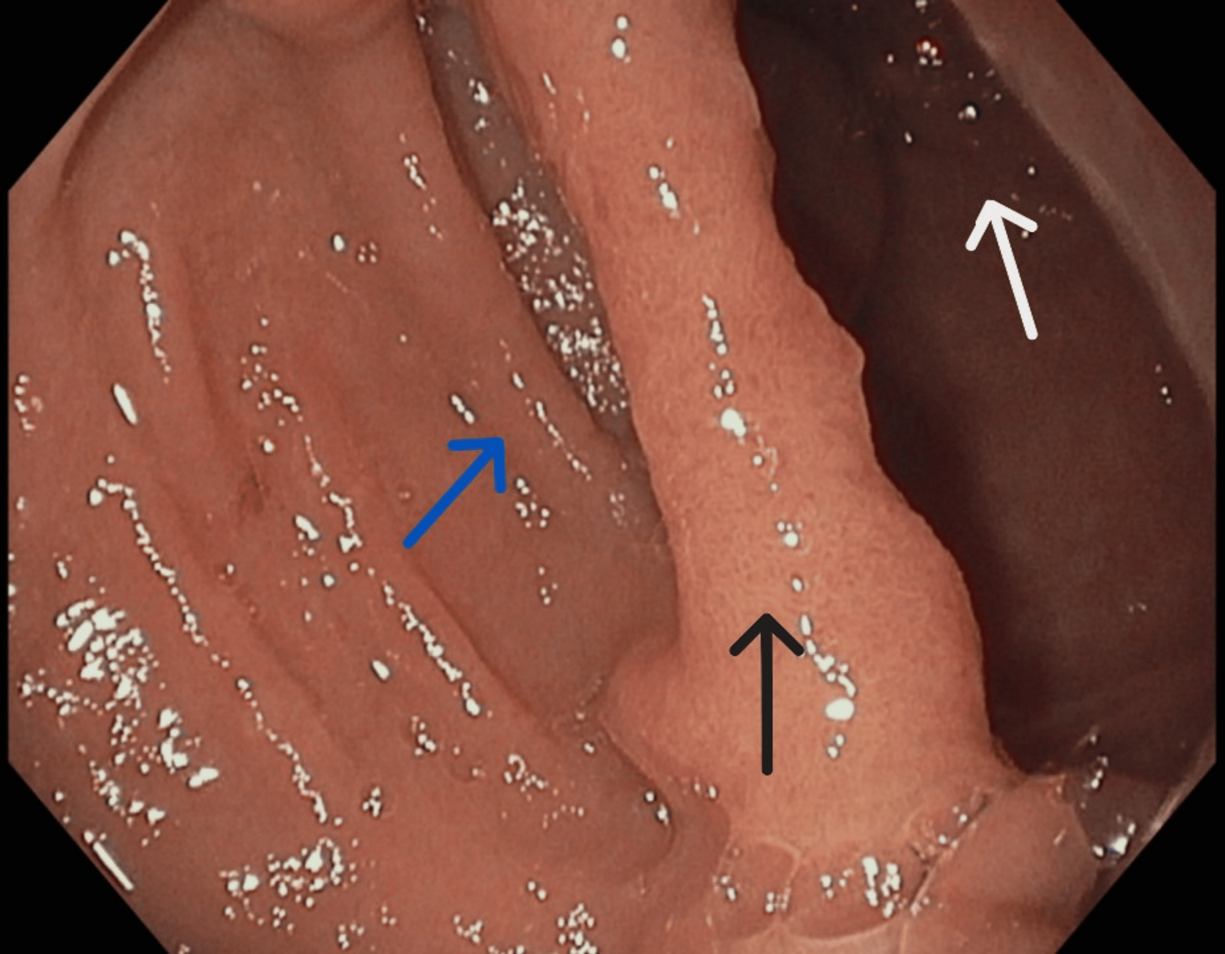
Endoscopic image of the base of the intraluminal duodenal diverticulum (black arrow), seen in the middle of the image. The proximal afferent origin (blue arrow) is partially visualized on the left side of the image, and the true lumen of the second portion of the duodenum (white arrow) on the right side.

**Figure 4:**
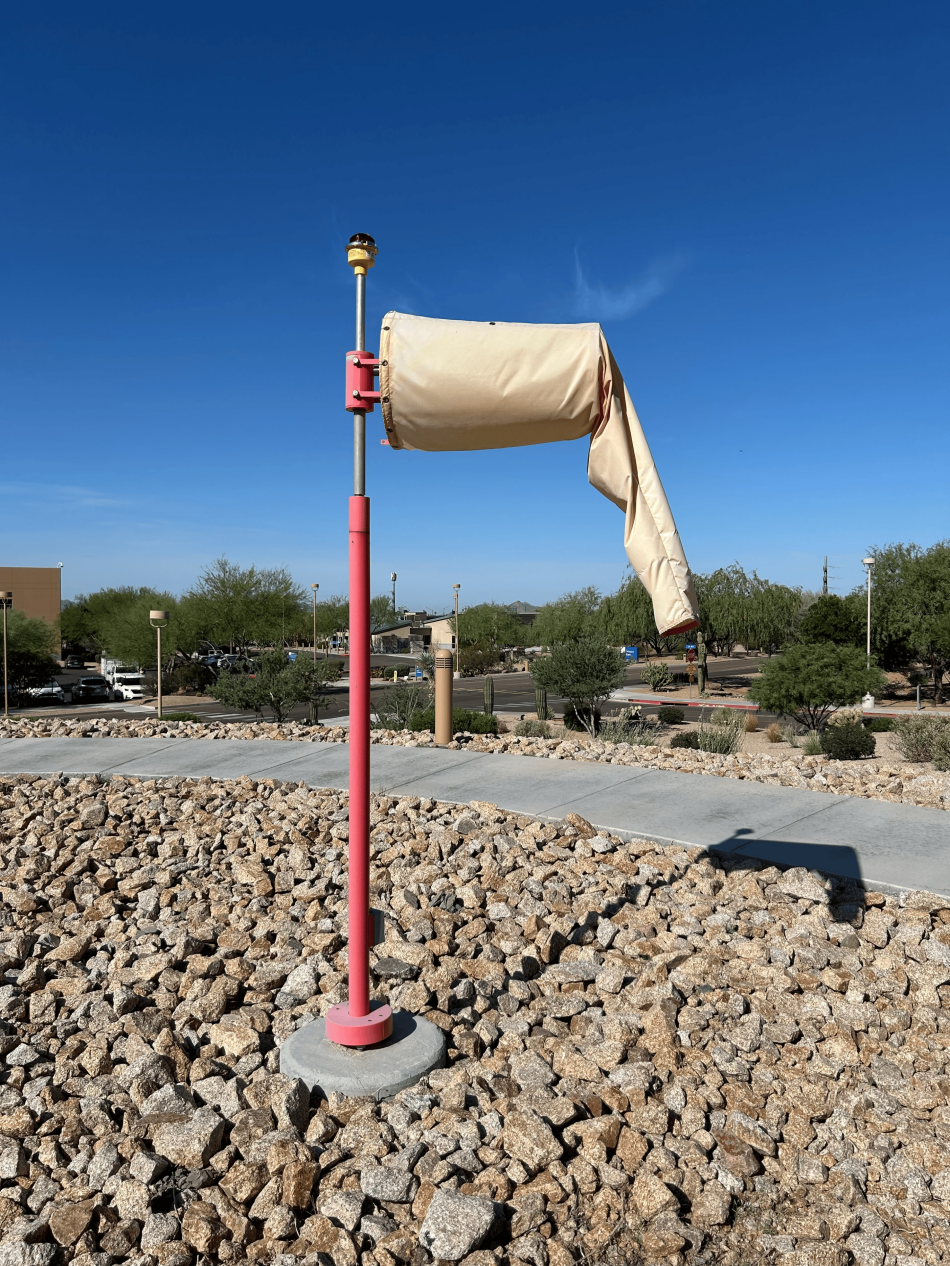
An aviation-grade windsock located adjacent to a helicopter landing pad near a hospital emergency department. Image credits: Author's own

A 76-year-old woman with no known congenital anomalies and a medical history of appendiceal adenocarcinoma status post partial right hemicolectomy with ileocecal valve sparing, mixed connective tissue disease, and hypertension presented to the gastroenterology clinic with a chief complaint of chronic, intermittent diarrhea. She endorsed passing three to five stools daily that were Bristol stool type 5 or 6, for approximately a year. She denied any weight loss, postprandial pain, obstructive gastrointestinal symptoms, nocturnal stooling, melena, hematochezia, tenesmus, or steatorrhea. Previous trials of over-the-counter loperamide modestly decreased her stooling frequency. Serologic testing of anti-tissue transglutaminase IgA, total IgA, and thyroid-stimulating hormone was normal. There was no evidence of anemia. A review of a recently performed computed tomography scan of the abdomen and pelvis was unremarkable. The patient was scheduled for bi-directional endoscopy. During EGD, we discovered an inverted IDD. The IDD arose from the medial wall of the second portion of the duodenum, just proximal to the major papilla (Figures 1-3). The aforementioned IDD has been described as a 'windsock diverticulum', as it structurally resembles an aviation-grade windsock (Figure 4). We elected not to biopsy the IDD due to the absence of any endoscopically inflamed, ulcerated, or otherwise malignant-appearing tissue. Without symptoms of duodenal or definitive papillary obstruction, diverticulotomy or diverticulectomy was not pursued. Duodenal mucosal biopsies were negative for celiac sprue, Whipple's disease, or Giardia. Duodenal aspirates were negative for small intestinal bacterial overgrowth. Colonoscopy showed no gross endoscopic abnormalities and revealed a competent ileocecal valve with normal-appearing ileal mucosa. Colonic mucosal biopsies were negative for inflammatory or microscopic colitis.

## Discussion

Our clinical vignette describes an uncommon endoscopic finding incidentally discovered during EGD in a patient who presented with a chief complaint of diarrhea. Testing for thyroid dysfunction, celiac disease, small intestinal bacterial overgrowth, and inflammatory and microscopic colitis was negative. Prior cases describe patients with IDD presenting with obstructive gastrointestinal symptoms, pancreatitis, or bleeding [3-4]. In the case of our patient, the IDD could not be definitively labeled as the etiology of her diarrhea. Histologically, IDD tissue is substantially vascular, but there have been no cases published that suggest potential malignant transformation. Gastroenterologists have used snare diverticulectomy and endoscopic submucosal dissection scissors or knives for diverticulotomy. Immediate and delayed bleeding may occur following removal, requiring hemostatic clip deployment or electrocautery, so close observation is recommended post-intervention [5]. In patients with acute small bowel obstruction, laparoscopic partial duodenectomy may be required [6]. Because of the relatively mild nature of her diarrhea symptoms and the fact that no causal relationship could be established, the patient did not elect to undergo subsequent interventional EGD to have the IDD removed. She elected to manage her symptoms with over-the-counter loperamide and dietary modifications. She will be monitored for symptoms of post-prandial duodenal obstruction, pancreatitis, and bleeding, and we will consider the IDD as a possible cause of her diarrhea, should it persist or worsen with no other obvious cause.

## Conclusions

Recognizing the characteristic appearance of IDD as a benign anomaly seen during EGD may allow care teams to avoid unnecessary cross-sectional or endoscopic ultrasound imaging. Though this is a single case report, merely observing and documenting the presence of an IDD may suffice as morbidity, as possible complications from endoscopic resection or surgical intervention can occur. Individual clinical judgement, multidisciplinary team discussion, and patient preference should take precedence when discussing the possibility of IDD removal versus observation.
